# Serious Adverse Events after a Single Shot of Intrathecal Morphine: A Case Series and Systematic Review

**DOI:** 10.1155/2022/4567192

**Published:** 2022-03-10

**Authors:** Mark V. Koning, Elmer Reussien, Beatrijs A. N. Vermeulen, Svenja Zonneveld, Elsbeth M. Westerman, Jurgen C. de Graaff, Bernard M. Houweling

**Affiliations:** ^1^Department of Anesthesiology and Critical Care, Rijnstate Hospital, Arnhem, Netherlands; ^2^Department of Anesthesiology, Erasmus MC, Academic Medical Center Rotterdam, Rotterdam, Netherlands; ^3^Department of Anesthesiology, Haaglanden Medical Center, The Hague, Netherlands; ^4^Department of Clinical Pharmacy, Haaglanden Medical Center, The Hague, Netherlands

## Abstract

**Background:**

The dose of intrathecal morphine is important because of its narrow therapeutic range. Due to a compounding error, pharmacy-compounded, ready-to-use syringes contained 1 mg ml^−1^ morphine instead of the intended 50 mcg ml^−1^. Six patients consequently received this twenty-fold dose. This study aims to describe the serious adverse events in these six patients and a systematic review is added to describe the characteristics of serious adverse events after intrathecal morphine.

**Methods:**

A retrospective case series described all six patients that received the erroneous morphine intrathecally for analgesia after laparoscopic segmental colonic resections. The patients' charts were reviewed for the occurrence, timing, duration and management of adverse events, the vital signs at the night after surgery, and length of hospital stay. A systematic review investigated characteristics of serious adverse events after intrathecal morphine in a perioperative setting.

**Results:**

Four patients had a serious adverse event, which was respiratory depression combined with somnolence (*n* = 3) and hypotension (*n* = 1). The review yielded 63 cases with serious adverse events, predominantly somnolence and/or respiratory depression. The onset occurred between 2 and 24 hours after injection. The severity of symptoms varied and life-threatening respiratory depression only occurred after a dose >900 mcg or when potentiating medication was used. Naloxone did not affect analgesia. No prolonged sequalae occurred.

**Conclusion:**

This study reveals that respiratory depression and somnolence are the predominant serious adverse events after intrathecal morphine in a perioperative setting and demonstrated a large variation in the presentation of symptoms.

## 1. Introduction

Intrathecal morphine is known to provide effective postoperative analgesia since 1979 [1]. Due to its hydrophilic properties, intrathecally administered morphine remains in the cerebrospinal fluid for a prolonged period of time, which results in a duration of action up to 36 hours, but simultaneously carries the risk of a delayed respiratory depression [2–4]. Several studies suggest a dose-dependent incidence of delayed respiratory depression [5–7]. The incidence of respiratory depression after a dose of less than 1 mg intrathecally administered morphine varies between 0.5 and 3.0% in studies including 492 and 5705 patients [5,6,8–11]. This variance in the incidence is for an important part caused by the heterogeneity in definition of respiratory depression [4].

This heterogeneity in definition also impairs the interpretation of the severity of respiratory depression. It varies amongst studies with criteria including low respiratory rate, hypercapnia, and hypoxemia [4]. Life-supporting therapy is seldom required [8]. Furthermore, the onset, duration, accompanying symptoms, and risk factors are also not fully elucidated. Amongst the accompanying symptoms is somnolence of interest because it is associated with an increased risk for respiratory depression [12]. These characteristics require further clarification in order to interpret the clinical importance and provide a basis for recommendations for precautions or monitoring [13].

Recently, our hospital initiated the routine use of intrathecal morphine as postoperative analgesia for patients undergoing laparoscopic segmental colonic resections, based on the positive results of several studies [14–16]. Pharmacy-compounded, ready-to-use syringes of 2.5 mg mL^−1^ bupivacaine and 50 mcg mL^−1^ morphine were used for this purpose. Unfortunately, due to a human error during the pharmaceutical compounding process by an external compounding pharmacy, the morphine concentration was 1000 mcg mL^−1^ instead of the labelled 50 mcg mL^−1^. Consequently, a total of six patients accidentally received 3000 to 5000 mcg morphine intrathecally, until this error was discovered after a critical review of four cases with serious adverse events. A retrospective review of these patients who received a twenty-fold dose of intrathecal morphine offers a unique opportunity to describe the severity of the adverse events after intrathecal morphine.

This study aims to elucidate the severity and characteristics of serious adverse events after the administration of intrathecal morphine, which may guide recommendations for monitoring. Our study has two methods: firstly, a retrospective case series describes the severity and characteristics of adverse events. Secondly, a systematic review of the literature for individual cases of serious adverse events after the use of intrathecal morphine in a perioperative setting aims to describe the onset, the duration, accompanying symptoms, and the severity and risk factors.

## 2. Materials and Methods

We performed a retrospective study of all six patients who have received intrathecal morphine between May 31, 2019, and June 7, 2019, in a teaching hospital in the Netherlands (Haaglanden, The Hague). All patients received medication from the same batch of erroneously pharmacy-compounded syringes and no medication of this batch was administered to other patients. The study protocol was approved by the local hospital ethics committee (Leiden-Den Haag-Delft G20.094) because patients were not subjected to investigational actions. All patients provided informed consent for the anonymous use of their data. The data were retrieved from the electronic hospital medical records (HIX 6.1, ChipSoft, Amsterdam, The Netherlands).

### 2.1. Medication Error

The use of intrathecal bupivacaine/morphine as method of analgesia was initiated on the first of May 2019. The hospital pharmacy provided the aseptically compounded medication with 5 ml syringes labelled to contain an isobaric concentration of 2.5 mg ml^−1^ bupivacaine and 50 mcg ml^−1^ morphine. The syringes from the erroneously prepared batch were available for clinical use from the 31st of May 2019 until the decision to stop this method of analgesia on the 7th of June 2019. During that time, no other batch of bupivacaine/morphine was concurrently used. On the 7th of June 2019, it was noted that four patients had serious side effects, which were associated with an intrathecal high dose of morphine in a short period of time. The medication was recalled to the pharmacy department on June 12, 2019. All but six syringes were sent to the pharmacy department, since these six have been used for patient care. Analysis showed that the ready-to-use syringes contained 2.5 mg mL^−1^ bupivacaine and 1000 mcg mL^−1^ morphine. A human error in the compounding process and no routine in the process of drug concentration analysis before release of the batch were identified as causes for this medication error. The aseptically compounded admixture was based on commercially available morphine ampoules and bupivacaine vials. In retrospect, from a risk reduction perspective, the batch of thirty ready-to-administer syringes should have been subjected to drug concentration quality control. It was reported to the patients and the authorities and practice has changed according to the recommendations of the authorities. One of which is routinely performing drug concentration quality control in aseptically prepared syringes in small batches.

### 2.2. Standard Care

Intrathecal bupivacaine/morphine was exclusively administered to patients undergoing laparoscopic segmental colonic resections. On the day of surgery, patients were admitted to the hospital and no sedative premedication was routinely administered.

Spinal anesthesia was administered through a 27-G pencil point needle (Pencan, Braun, Melsungen, Melsungen, Germany) in a sterile manner. Per local protocol, 3–5 mL of a ready-to-use syringe produced by an external compounding pharmacy as described before was administered for spinal anesthesia in the operation room. The dose of intrathecal morphine was at the discretion of the anesthesiologist and in the local protocol that has recommended to use 4–5 mL (i.e., 200–250 mcg of morphine) for patients under 71 years of age, while 3–4 mL (i.e., 150–200 mcg of morphine) was used for elder patients. Thereafter, general anesthesia was administered at the discretion of the anesthesiologist using a combination of an opioid (sufentanil or remifentanil), an anesthetic agent (propofol or sevoflurane), and neuromuscular blockade (rocuronium). A urinary catheter was inserted before surgery commenced.

During surgery, 1000 mg of paracetamol, 75 mg of diclofenac, 4 mg of dexamethasone, and 4 mg of ondansetron were routinely administered. Intravenous fluids were targeted at a fluid balance of less than 750 ml surplus. Administration of phenylephrine and norepinephrine as vasopressors was at the discretion of the attending anesthesiologist. At the end of surgery, a train-of-four-ratio-measurement was routinely performed to measure residual neuromuscular blockade. If necessary, this was antagonized with sugammadex. If the patient was arousable and protective airway reflexes and sufficient spontaneous ventilation were present, the trachea was extubated, and the patient was transferred to the Post-Anesthesia Care Unit (PACU).

At the PACU, 2.5 mg piritramide was administered intravenously by a nurse if pain scores were >4 on a Numeric Rating Scale (NRS). Additional ondansetron or 0.6–1.2 mg dehydrobenzperidol was administered for nausea or pruritus if needed at the discretion of the anesthesiologist. The patient was discharged to the ward when pain scores were <4 NRS, no nausea or other side effects were present, and the Aldrete score was >8.

At the ward, postoperative analgesia was continued with 1000 mg paracetamol four times daily, naproxen 250 mg three times daily, and short-acting oxycodone 5 mg if needed. Up to three doses of 4 mg ondansetron and 1 mg haloperidol were allowed for nausea and pruritus if needed. Heart rate, noninvasive blood pressure, pulse oximetry, respiratory rate, and consciousness were routinely measured at least every 4 hours. Three-liter min^−1^ of oxygen was routinely administered by nasal cannula and adjusted to maintain a SpO_2_ > 94%. Based on a Modified Early Warning Score (MEWS), the nurses consulted the attending physician and/or the Intensive Care Unit if the score was higher than 3 [17]. The house officer and intensive care physician were 24 hours per day available and usual response time was within 5 minutes. The choice to call the house officer or the intensive care physicians depended on the severity and judgement of the attending nurse. The attending nurse could call the intensive care physicians directly without consulting the surgical resident first.

### 2.3. Data Collection

Patient characteristics, all administered drugs on the day of surgery and first postoperative day, duration of surgery, duration of anesthesia, time spent on the PACU, and worst vital signs measured on the ward were noted. The patient's chart was investigated for any adverse events during hospital admission and on the first outpatient visit. In case of an adverse event, time and symptoms of this adverse event were registered, as well as the undertaken diagnostic measures, the treatment, and duration of treatment.

### 2.4. Systematic Review of Literature

In order to elucidate the severity and characteristics of the adverse events after intrathecal morphine, a literature search for these outcomes was performed in March 2021. The registration was declined by Prospero for not meeting the inclusion criteria. The databases Medline, Embase, Cochrane CENTRAL, and Web of Science were used. No language restrictions applied. The search combined the terms “Intrathecal” OR “Spinal” AND “Morphine” and excluded the terms “Chronic”, “Pump”, or “Caesarean Section” (see supplementary data file A for search strategy). Duplicates were removed using Endnote. The bibliographies from selected studies were screened for relevant studies. The cases of the current case series were included as well.

Because the review aimed to investigate characteristics of serious adverse events, individual case data were required. Therefore, inclusion criterium was any individual case description of one or more patients with an adverse event after the use of a single shot of intrathecal morphine in a perioperative setting. All types of studies were included and data from cohort studies were only used when individual patient data were identifiable. Exclusion criteria were aggregate data, obstetric cases, and intrathecal morphine administered through an intrathecal pump or for chronic use. The latter two exclusion criteria were based on the reason that obstetric and non-opioid-naïve patients are likely to have a different response than opioid-naïve surgical patients. In addition, the time of onset and dose are difficult to determine after continuous infusions and postoperative patients may have other causes of hypoxemia compared to outclinic patients, such as atelectasis.

Two authors (MVK and BMH) screened the titles and abstracts. A study was included for a full-text analysis when at least one author deemed it of interest. Both investigators (MVK and BMH) independently read the full texts of these citations. Data extracted from the full text was the author, year of publication, age and gender of the patient, type of surgery, dose of intrathecal morphine, the concomitant use of other intrathecal medication, the use of sedative medication on the day of surgery, the presenting symptoms, the time between injection and symptoms, the duration of the symptoms, the use and dose of antagonizing agents, the vital signs (blood pressure, heart rate, respiratory rate, pCO_2_, pO_2_, SpO_2_, and consciousness) upon presentation, the use of mechanical ventilation, or other types of life support and the overall outcome.

### 2.5. Outcomes of the Systematic Review

All cases were screened for the criteria of serious adverse event, defined as an impaired vital sign, including either somnolence, defined as a Glasgow Coma Scale <14 or described as “difficult to arouse,” respiratory depression, defined as a respiratory rate below 10 breaths per minute, a PaCO2 > 7.0 kPa or an SaO2 < 90%, or cardiovascular instability, defined as a heart rate <50 bpm or >120 bpm or a mean arterial blood pressure <55 mmHg or >110 mmHg. The cases were also screened for a life-threatening respiratory depression, which was defined as a respiratory rate <4 breaths per minute, a SpO2 <85% or other signs of hypoxemia, or a PCO2 > 8 kPa.

### 2.6. Statistics

Data are presented as median (interquartile range) [minimum-maximum] or as *N* (%) when applicable.

## 3. Results

### 3.1. Case Series

A total of six patients (age 63–80, 3 female, 3 male) received intrathecal analgesia with medication from the erroneous batch (Table 1). Four patients had serious adverse events, which were respiratory depression combined with somnolence (*n* = 3) and hypotension (*n* = 1). In the other two patients, no events were detected and recovery from surgery appeared uneventful. These two patients had uneventful intrathecal injection and signs of an intrathecal block due to the bupivacaine. None of the patients required any additional opioids after surgery. All four cases with serious adverse events are described in the supplementary data file B. Respiratory depression consisted of hypoxemia (Patient 4), hypercapnia (Patients 1, 4, and 6), and a respiratory rate <10 breaths per minute (Patients 1 and 4). Hypercapnia was present in all three patients in whom CO_2_ was measured. In all three patients, the respiratory depression resolved after the administration of supplemental oxygen and naloxone; none of the patients required mechanical ventilation. One patient was admitted to the Intensive Care Unit (patient 4) and 2 patients were treated on the ward.

These three patients (patients 1, 4, and 6) also suffered from somnolence. The onset time differed, since patient 4 was immediately unarousable after a 2-hour surgery and the other two patients were somnolent after 5 (patient 1) and 13 hours (patient 6) after intrathecal injection, respectively. Patient 4 received alprazolam prior to surgery, although patient 3 received midazolam prior to surgery and did not suffer from somnolence (Table 1). All cases of somnolence were accompanied with respiratory depression.

Patient 3 was hypotensive without tachycardia after a period of normotension. This patient had no signs of sedation or respiratory depression and showed no signs of hemorrhage, hypovolemia, or other causes of shock. He was already admitted to the ICU (Intensive Care Unit) because of comorbidities as planned preoperatively. A continuous infusion of norepinephrine for 24 hours resolved the hypotension. All other patients did not have any hemodynamic consequences.

The serious adverse events occurred between 2 and 20 hours after injection and lasted from 1 to 37 hours (Table 1). No patients received additional opioids in the postoperative phase. All six patients survived and did not sustain any permanent damage related to adverse event caused by the intrathecal morphine.

### 3.2. Review of Literature

**Figure 1:**
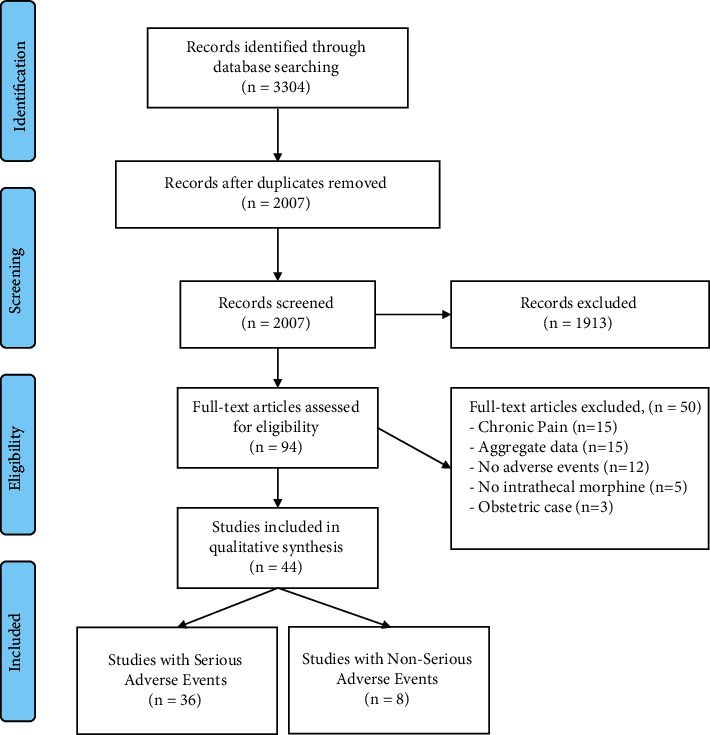
Flow diagram of the systematic review.

The search obtained 2007 articles, from which 1903 studies were excluded after screening of abstracts. The flow diagram is presented in Figure 1 and the search strategy is attached in the supplementary data file A. From the remaining 104 studies, 44 papers were included and yielded 68 case descriptions of adverse events after the use of intrathecal morphine. With the inclusion of the data from the current case series, 72 cases were analyzed (see supplementary data file C). Sixty-three patients had disturbed vital signs and were deemed as a serious adverse event related to morphine. The deemed unrelated or nonserious adverse events (*n* = 9) consisted of nystagmus (*n* = 2), fascia dehiscence after lumbar surgery (*n* = 2), meningitis (*n* = 1), hypothermia and/or diaphoresis (*n* = 3), and pain (*n* = 1). Two patients deemed as a serious adverse event also had accompanying adverse events, which were a nystagmus and a Transient Ischemic Attack (supplementary data file C). The TIA was believed to be caused by hypercapnia, leading to an intracerebral steal phenomenon.

**Figure 2:**
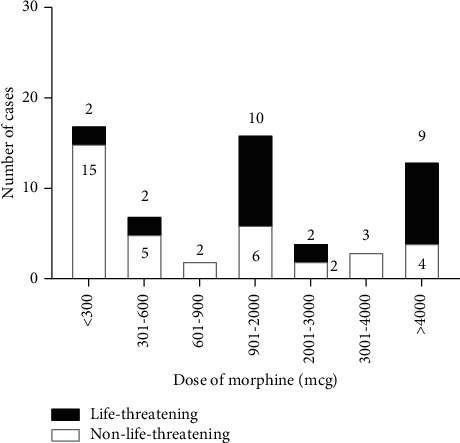
The number of reported cases with somnolence and/or respiratory depression, reported per range of intrathecal morphine dose.

The sixty-three serious adverse events were classified as somnolence (*n* = 28), respiratory depression (*n* = 54), and hypotension with relative bradycardia (*n* = 2). Eighteen patients were both somnolent and respiratory depressed.  Table 2 displays the characteristics of the cases with somnolence, respiratory depression, and hypotension. The administered dose in the cases with respiratory depression and/or somnolence is displayed in Figure 2. It demonstrates SAE occurs after all doses. The distribution suggests that more severe cases were associated with the higher doses of intrathecal morphine because 15% of the patients with a dose below 900 mcg of intrathecal morphine had a life-threatening respiratory depression, compared to 58% of the doses over 900 mcg. Accidental overdosing was reported in two studies [18,19]: one study administered morphine at the level of T3 after a spinal tap for epidural analgesia [20] and one study reported manual dilution of the morphine [21]. No fatalities were reported.

*Somnolence* was often described as “difficult to arouse.” More specific data are displayed in Table 2. Treatment consisted of naloxone (*n* = 23, 82%), which was repeatedly or continuously administered in five cases. In one case, cerebrospinal fluid irrigation was performed [22] and another case resolved after the administration of flumazenil since benzodiazepines were coadministered [21]. The duration of somnolence was infrequently reported, but either resolved after a continuous naloxone administration or spontaneously after 12–24 hours. Somnolence was combined with respiratory depression in 19 cases, which was 68% of all the cases presenting with somnolence.

*Respiratory depression* was reported in 54 cases and the definition varied from an elevated PaCO_2_> 6.6 kPa to a respiratory rate below 10 breaths per minute. The reported respiratory rates during the symptoms varied between apnea and 16 breaths per minute. CO_2_ levels varied between 6.8 and 15.1 kPa. Respiratory depression was combined with somnolence in 19 cases, which was 33% of all the cases presenting with respiratory depression. Thirty-eight patients were antagonized with naloxone, which was repeatedly or continuously administered in 8 patients. Twelve patients (23%) received ventilatory support, although often patients were already ventilated as part of routine postoperative cardiosurgical care or before administration of naloxone. Naloxone resolved the respiratory depression in all but two cases. Sidi et al. noted no effect of 400 mcg naloxone after 4000 mcg of intrathecal morphine and ventilated the patient mechanically [23]. Krenn et al. noted no effect on respiratory depression of 800 mcg naloxone after 100 mcg of intrathecal morphine, even though other nonserious effects resolved [24]. The patient required no ventilatory support and the respiratory depression resolved spontaneously.

The criteria of *life-threatening respiratory depression* were met in 25 cases. Four cases recovered spontaneously without the need for therapy or life support. Another four patients were mechanically ventilated and 19 patients were antagonized. The criterium of hypoxemia was reported in our case after 4000 mcg morphine and six other cases appeared “centrally cyanotic” after 10,000–15,000 mcg morphine [18,25,26]. A respiratory rate <4 breaths per minute was reported in 7 cases after 3000–15,000 mcg morphine [18,23,27,28] and in one patient with a Cheyne-Stokes breathing pattern after 100 mcg morphine [24]. The remaining cases met the criterium of a pCO2 > 8 kPa. Benzodiazepines were administered concomitantly in some of these cases and flumazenil was required as well to antagonize the respiratory depression in one case [21].

Four cases received a dose less than 900 mcg of intrathecal morphine, yet still had life-threatening respiratory depression (Figure 2). Two of these cases had co-administration of benzodiazepines and in one case flumazenil was administered and resolved the respiratory depression [21]. The other case subsided spontaneously [29]. The third case received continuous IV fentanyl postoperatively [30]. In the fourth patient, naloxone resolved the nausea, but not the Cheyne-Stokes breathing [24]. No ventilatory support was required in this patient. Since the patient received granisetron, ondansetron and metoclopramide for the nausea, the authors hypothesized that the opioid analgesia was potentiated through a dopamine-2-receptor antagonism [24].

*Hypotension* without tachycardia was reported in two cases, several hours after the intrathecal injection. One case was reported in the present case series (see supplementary data file B); another case involved a female patient who received 400 mcg of intrathecal morphine in 5% dextrose, combined with 40 mg of lidocaine for an orthopedic procedure [31]. Several hours later, she had a respiratory rate of 6 bpm, a heart rate of 46 beats per minute, and an arterial blood pressure of 70/50 mmHg. All her vital signs improved after a single dose of 400 mcg naloxone.

## 4. Discussion

This case series showed that an unintentional high dose of intrathecal morphine causes symptoms with a variety of severity and timing. The review of the literature showed that somnolence and respiratory depression were the most frequently reported symptoms. These symptoms were often coexisting, although both could also appear separately. The onset time of somnolence and respiratory depression occurred between 2 and 24 hours after injection. The dose of morphine associated with adverse events varied. Doses less than 900 mcg of morphine resulted in respiratory depression, but this was only life-threatening when potentiating medication was co-administered. Life-threatening adverse events were associated with doses over 1000 mcg of morphine. All but two patients responded well to naloxone and prolonged administration was sometimes necessary. None of the patients had prolonged sequalae due to the adverse effects of intrathecal morphine.

Somnolence and respiratory depression often coincide, but only one of these symptoms was present in 62% of the reported cases. Possibly respiratory depression was not of sufficient severity or duration to cause somnolence. However, somnolence also occurred without signs of respiratory depression, which is in line with the finding that distinct pathways are involved for both symptoms [32]. Our results did not identify a factor that differed between patients that were either somnolent or respiratory depressed after intrathecal morphine. Two patients in our case series received benzodiazepines; one of them had no signs of respiratory depression. Possibly, the sedative effects may have worn off in the patient without respiratory depression.

Our analysis included two cases of hypotension with a relative bradycardia, but uncertainty remains if this was caused by intrathecal morphine. Hypotension is rarely reported as a consequence of intrathecal morphine, yet experimental data shows that intrathecal morphine can exert a hypotensive effect [33,34]. All other patients included in the current review remained normotensive. Actually, most patients with hypercapnia are relatively hypertensive and tachycardic [35]. In our case series, the patient had comorbidities that could contribute to hypotension as well. This adds to the uncertainty to conclude that the hypotension in these cases is a consequence of the intrathecal morphine.

This review also demonstrates that the onset of symptoms may not always be delayed, but may also be present immediately after surgery. The latter is unexpectedly fast, considering that the morphine injected at a lumbar level requires rostral spread to reach a supraspinal level [3]. According to the theory of rostral spread in the cerebrospinal fluid (CSF), the onset of symptoms should be dependent on CSF volume of distribution, the distance to cerebrum, or CSF flow. Volume of distribution and distance to the cerebrum could be related to the patients' height, but no correlation between onset and the patients' height was found in our case series. CSF flow depends on CSF production, ciliary function, respiratory rate, and heart rate [36]. Especially inspiration is a major contributor, which may affect onset [37]. The current review was unable to determine this effect. In addition, we cannot exclude spinal pathology, such as spinal stenosis. Some authors suggested that baricity of morphine when dissolved in dextrose combined with the position of the patient may result in an earlier onset of respiratory depression [23,31], but we were unable to demonstrate this because an early onset also occurred with the use of isobaric morphine.

The severity of respiratory depression in the literature varies from a mildly elevated CO_2_ to a life-threatening hypoxemia or apnea [4]. We assume that the cases found in our review of the literature are likely the worst cases because mild cases may not be detected or published. Our review found twenty-five cases that may be regarded as life-threatening. None of these cases resulted in a fatality. These cases were all after >900 mcg of morphine [18,23,25–28,38–40] or after low dose of morphine with coadministration of potentiating medication [21,24,29,30]. The latter consisted of one case of 100 mcg of self-diluted, intrathecal morphine and 3 mg of IV midazolam, in which flumazenil resolved the respiratory depression [21], one case in which 400 mcg of intrathecal morphine and 4 mg of IV lorazepam resulted in a pCO2 of 8.5 kPa which resolved spontaneously [29], one case with concomitant continuous IV fentanyl [30], and one case in which 100 mcg of intrathecal morphine combined with intravenous metoclopramide and granisetron resulted in a Cheyne-Stokes breathing pattern (CSBP) [24]. This CSBP may not be related to morphine because it did not respond to naloxone and CSBP is unlikely to be caused by opioids [41]. Furthermore, no further life support or treatment was installed for this CSBP, which downgrades its severity [24]. In these four cases with coadministration of potentiating medication, it is questionable if the life-threatening respiratory depression is caused solely by intrathecal morphine.

Respiratory depression may result in hypoxemia. Hypoxemia caused by opioid-induced respiratory depression results from a decreased alveolar pO_2_ due to the hypercapnia, as depicted by Dalton's law. A small increase in FiO_2_ reverses this type of hypoxemia, as long as there is no increase in A-a gradient. To avoid the hypoxic consequences of respiratory depression, we suggest to routinely administer 3 L min^−1^ of supplemental oxygen after the use of intrathecal morphine. In addition, clinicians should investigate and treat the cause of respiratory depression and hypoxemia when present.

Treatment with relatively low-dose naloxone antagonized the adverse effects in all but two patients while analgesic effects remained. A concomitant effect of potentiating medication is not excluded in these two cases [23,24]. It is known that analgesic effects remain, while respiratory depression and somnolence are reversed with naloxone after neuraxial morphine [42]. A proposed mechanism of this phenomenon is that the morphine concentration is the highest around the injection site, which is the substantia gelatinosa in the lumbar region [43]. This higher concentration of morphine would require a higher concentration of antagonist, compared to the lower concentrations of morphine in the respiratory center. Based on this differential effect, an approach can be to administer continuous naloxone routinely to prevent respiratory depression but maintain analgesia [44,45]. However, this strategy requires further research regarding effectiveness and side effects.

This paper partially contradicts the recommendations from the American Society of Anesthesiologists, which recommends monitoring respiratory rate, oxygenation, and level of consciousness every hour for the first 12 hour and every 2 hours for 12 hours thereafter [13]. First, our case series demonstrates that the onset may be sudden, which questions the ability of an hourly monitoring to detect an adverse event. Second, such a strategy is likely to be inefficient, since the majority of the cases are not life-threatening, which is supported by the data from Gwirtz et al. [8]. Third, such a strategy adds nursing workload, which may be unfeasible or limit the use of an effective analgesic technique. One could argue to avoid the use of intrathecal morphine in such a situation, although Gehling and Tryba noted that changing intrathecal to intravenous PCA opioids does not reduce the risks, which is supported by an experimental study [5,46]. However, the cases that were life-threatening all had co-administration of potentiating medication such as benzodiazepines or used doses over 900 mcg morphine. Furthermore, the incidence, timing, and severity of respiratory depression are comparable to systemically administered opioids per Patient Controlled Analgesia (PCA)-pump [47–49]. Therefore, the results of the present study suggest avoidance of co-administration of benzodiazepines, avoiding doses over 900 mcg of intrathecal morphine and applying the same monitoring as for PCA-administered opioids in combination with the previous mentioned routinely administration of supplemental oxygen. Alternatively, if potentiating medication is required, an intensive monitoring strategy with continuous monitoring of vital signs is warranted for 24 hours.

This study has several limitations to consider. First, the review does not provide incidences of adverse events after the use of intrathecal morphine. For that piece of information, an observational study of Gwirtz et al. regarding 5969 patients using 200–800 mcg of intrathecal morphine remains the most robust study, which detected an incidence of 3.0% of respiratory depression, defined as a respiratory rate <10 bpm or a pCO_2_ > 50 mmHg [8]. Of these patients, none had life-threatening respiratory failure [8]. Other studies with various doses of intrathecal hydrophilic opioids found incidences of 3.0% of 1022 patients [9], 1.5% of 1039 patients [10], 1.0% of 492 patients [11], 3.0% of 327 patients [5], and 0.5% of 944 patients [6], with varying definitions of respiratory depression. Second, the majority of the data of this review resulted from case reports, which are inherently retrospective of nature, just as our case series. This leads to missing data, as exemplified by our case series in which pCO_2_ was only determined in three patients. If in all patients pCO_2_ had been determined, possibly more patients could have been defined with respiratory depression. In addition, observational and randomized trials were screened and included in this analysis, but these contributed little because individual patient data were seldomly reported. Third, two patients we classified as asymptomatic in our case series might have been truly asymptomatic, or the symptoms were not detected in our clinical setting, which might result in a false sense of safety. Fortunately, it did not lead to permanent adverse sequelae. This applies to the review as well, since patients that were classified as asymptomatic might be symptomatic but undetected. Fourth, a review is inherently limited by publication bias. One can argue that the most severe cases are likely to be reported, although it is not excluded that a clinician is reluctant to report an inadvertent fatal case. Fifth, this review of the literature did not include adverse events related to intrathecal morphine in patients with intrathecal infusion pumps or in patients treated for chronic pain. We believe that such a clinical setting is not transferable to adverse effects of a single shot of intrathecal morphine in a perioperative setting, unless a serious medication error occurs. Still, massive doses of intrathecal morphine may result in neurotoxicity, psychosis, myoclonias, epilepsy, allodynia, and motor block [50–57].

In conclusion, this paper showed that somnolence and/or respiratory depression were the most serious adverse events after a single dose of intrathecal morphine in a perioperative setting. In addition, this paper demonstrated that the onset and duration of symptoms vary greatly, making it unpredictable. Symptoms may occur after any dose of intrathecal morphine. The life-threatening cases of respiratory depression were associated with a dose over 900 mcg morphine or with concomitant use of potentiating medication. Naloxone antagonized the adverse effects in the majority of the cases without affecting analgesia. No patient had prolonged sequelae due to the use of intrathecal morphine. We suggest routine supplemental nasal oxygen, avoidance of high doses of intrathecal morphine, and concomitant use of potentiating medication in order to reduce the risk for life-threatening respiratory depression. Furthermore, drug concentration analysis of compounded admixtures in small batches is warranted, especially for the use of intrathecal morphine as demonstrated in this report.

## Figures and Tables

**Table 1:** Patients' characteristics.

Patient	Age-gender-type of laparoscopic surgery	Dose of ITM (mcg)	Time between ITM and symptoms (hours)	Duration of symptoms (hours)	Vital signs when symptoms were detected^	Class of SAE	Treatment	ICU admission	Length of hospital stay (days)
1	80-F-right hemicolectomy	5000	5	19	E3M6V5, 6 brpm, 7.1 kPa, 97%, 2 L min^−1^, 118/54, 71 bpm	S, RD	100 mcg naloxone, at 10 and 22 hours after ITM injection	No	8
2	73-M-rectosigmoid resection	5000				None		No	8
3	72-M-rectosigmoid resection	4000^*∗*^	4	24	E4M6V5, 17 brpm, 100%, 1 L min^−1^, 102/31, 54 bpm	H	Norepinephrine-infusion for 24 hours	Yes	4
4	74-F-right hemicolectomy	5000^*∗*^	2	36	E2M5V2, 8 brpm, 7.3 kPa, <70%, 0 L min^−1^, 100/65, 70 bpm	S, RD	Oxygen and naloxone-infusion for 36 hours	Yes	5
5	68-F-right hemicolectomy	3000				None		No	4
6	63-M-sigmoid resection	3000	13	6	E2M5V2, 12 brpm, 8.2 kPa, 97%, 1 L min^,−1^, 164/95, 94 bpm	S, RD	100 mcg naloxone	No	4

^*∗*^Received benzodiazepines preoperatively. ^Displayed as Glasgow Coma Score, respiratory rate, pCO_2_, SaO_2_, supplemental oxygen, noninvasive blood pressure, and heart rate. Bpm: beats per minute; brpm: breaths per minute; F: female; H: hypotension, ICU: Intensive Care Unit; ITM: intrathecal morphine, M: male, RD: respiratory depression, and S: somnolence.

**Table 2:** Data from the review of literature.

	Somnolence	Respiratory depression	Hypotension
*N*	28	54	2
Male (%)	11 (48%)	15 (38%)	1 (50%)
Age (years)	73 (63–78) [40–90]	72 (66–79) [40–90] (*n* = 20)	73[72–74]
Time of onset (h)	5 (2–6) [1.5–13] (*n* = 22)	6 (3–7) [1–24] (*n* = 35)	8 (4–12)
Dose (mcg)	2000 (250–5000) [60–15,000] (*n* = 27)	1000 (350–3000) [100–20,000] (*n* = 54)	2200 (400–4000)
Concomitant local anesthetics	16 (57%)	21 (40%)	2 (100%)
Systemic sedatives	7 (25%)	10 (19%)	2 (100%)
Naloxone	23 (82%)	38 (70%)	1 (50%)
Positive response to naloxone	22 (96%)	36 (95%)	1 (100%)
Dose of naloxone (mcg)	200 (100–200) [80–400] (*n* = 14)	300 (160–400) [80–1600] (*n* = 29)	400

Patients showing multiple symptoms are included in both groups. Missing data resulted in varying size of the groups and percentages are calculated over the reported data. Data is presented as *N* (%) or median (interquartile range) [range].

## Data Availability

Data will be shared by the corresponding author upon reasonable request.

## Supplementary Materials

Supplementary Materials A: search strategy. B: case descriptions. C: table of included studies.
