# Supplementary material for: Serious Adverse Events after a Single Shot of Intrathecal Morphine: A Case Series and Systematic Review
Source: Pain Res Manag. 2022 Mar 10;2022:4567192. doi: 10.1155/2022/4567192 (PMC8930253; doi:10.1155/2022/4567192)
Supplement: Supplementary Materials — A: search strategy. B: case descriptions. C: table of included studies. [file 4567192.f1.zip › 4567192.f1/Supplemental data file - cases.docx]

Supplemental data file

**Patient 1:**

A 80 year old female was scheduled for a laparoscopic right hemicolectomy. She had no contributing medical history and used no medication. Intrathecal anesthesia was administered with the use of a ready-to-use syringe containing 12.5 mg of bupivacaine and 250 mcg of morphine, which was 5000 mcg of morphine in reality. During general anesthesia with sevoflurane, 7.5 mcg of sufentanil was administered. Surgery ensued uneventful and lasted 80 minutes. The patient regained consciousness, the trachea was extubated and she was transferred to the Post Anesthesia Care Unit (PACU). She remained at the PACU for 1 hour and her vital signs remained normal. Five hours after intrathecal injection, the nursing staff was alarmed because of the somnolence and the slow respiration. Upon arrival, the attending physician detected no signs of airway obstruction, normal vital signs (NIBP 118/54, heart rate of 71 bpm, SpO2 97% with 2 L/min of nasal oxygen and respiratory rate of 12 breaths per minute) and a Glasgow Coma Scale of E3M6V5 after firm stimuli without signs of lateralization. The patient was re-evaluated 4 hours later by the same physician and no significant change was noted, other than a decrease in respiratory rate to 6 breaths per minute. An arterial bloodgas analysis showed a pCO2 of 7.1 kPa. 100 mcg naloxone was administered intravenously 10 hours after the intrathecal injection and the patient recovered to a normal conscious state and was without pain. She remained on the ward and somnolence re-occurred after two hours. No ICU admission was required as judged by the attending intensivist. The next morning she was too somnolent for mobilization and another 100 mcg naloxone was administered. No further repeat-dose was required. Later that first postoperative day, she was found to have an intra-abdominal hemorrhage based on a declining hemoglobin level and a repeat laparoscopy was needed. During induction of general anesthesia she aspirated and developed a pneumonia. She was discharged home 8 days after the first surgery.

**Patient 3:**

A 72 year old male was scheduled for a laparoscopic rectosigmoid resection. He had a history of hypertension, insulin-dependent Diabetes Mellitus, chronic renal failure for which he received intermittent hemodialysis, stroke and multiple myeloma. He did not use antihypertensive medication or beta-blocking agents. He was administered 5 mg of midazolam intravenously prior to the intrathecal injection for anxiety. Intrathecal anesthesia was administered with the use of a ready-to-use syringe containing 10 mg of bupivacaine and 200 mcg of morphine, which was 4000 mcg of morphine in reality. General anesthesia was maintained with sevoflurane and remifentanil. Surgery ensued uneventful and lasted 107 minutes. The patient regained consciousness, the trachea was extubated and he was transferred to the Intensive Care Unit, which was scheduled because of his comorbidities. Four hours after the intrathecal injection, his blood pressure dropped to 102/31 mmHg and his heart rate was 54 beats per minute. His respiratory rate was 17 breaths per minute and his consciousness was unaltered. He was continuously given norepinephrine for 24 hours, after which it could be stopped. No further sequalae occurred and he was discharged home 4 days after surgery.

**Patient 4:**

A 74 year old female was scheduled for a laparoscopic right hemicolectomy. She had a history of anxiety, for which she used 0.5 mg alprazolam as needed, which was the case one hour prior to surgery. Intrathecal anesthesia was administered with the use of a ready-to-use syringe containing 12.5 mg of bupivacaine and 250 mcg of morphine, which was 5000 mcg of morphine in reality. General anesthesia was maintained with sevoflurane and remifentanil. Surgery ensued uneventful and lasted 137 minutes. After the surgery, she was not arousable, but had an adequate spontaneous minute ventilation and protective airway reflexes. Her trachea was therefore extubated. Directly after extubation her oxygen saturation dropped to 70%, for which a nasal-pharyngeal airway was inserted and 5 L min^-1^ of oxygen per face mask was administered. One hour after surgery, her Glasgow Coma Score was still E3M5V2, although without signs of lateralization. At first, this was attributed to the alprazolam the patient received before surgery for severe anxiety. Four hours after the intrathecal injection, 400 mcg naloxone was administered, after which she was fully awake without pain. Shortly after she became somnolent again with a respiratory rate of 8 breaths per minute and was admitted to the ICU. At the ICU an additional dose of 400 mcg naloxone was administered, after which a continuous infusion was started. Continuation of this infusion was needed until the next evening (+36 hours after intrathecal injection). Initially, when the naloxone was tapered, she became somnolent and with hypopnea again. After the continuous infusion was stopped the next day, no signs of somnolence re-occurred. She was discharged home 5 days after surgery.

**Patient 6:**

A 63 year old male was scheduled for a laparoscopic sigmoid resection. He had no contributing medical history and used no medication. Intrathecal anesthesia was administered with the use of a ready-to-use syringe containing 7.5 mg of bupivacaine and 150 mcg of morphine, which was 3000 mcg of morphine in reality. During general anesthesia with sevoflurane, 15 mcg of sufentanil was administered. Surgery ensued uneventful and lasted 95 minutes. The patient regained consciousness, his trachea was extubated and he was transferred to the Post Anesthesia Care Unit (PACU). He remained at the PACU for 3 hours, predominantly for the treatment of nausea, for which two doses of 4 mg of ondansetron were provided. His vital signs remained within normal limits.

Thirteen hours after the intrathecal injection, during a meal on the ward, the patient was found unconscious. The Intensive Care Unit was consulted and upon arrival the patient was found less responsive, with food in his mouth, but with an unobstructed airway. His Glasgow Coma Scale was E2M5V2 without signs of lateralization. His vital signs were a heart rate of 94 bpm, blood pressure of 164/95, a SpO2 of 97% with 1 L/min of nasal oxygen, a respiratory rate of 12 breaths per minute and arterial pCO2 of 8.2. He was given 100 mcg of naloxone and within minutes he was fully awake without pain. He was observed on the ward and somnolence re-occurred during the four hours after ICU consultation, but not as severe as earlier. Further recovery went uneventful and he was discharged home 4 days after surgery.
