# Supplementary material for: Serious Adverse Events after a Single Shot of Intrathecal Morphine: A Case Series and Systematic Review
Source: Pain Res Manag. 2022 Mar 10;2022:4567192. doi: 10.1155/2022/4567192 (PMC8930253; doi:10.1155/2022/4567192)
Supplement: Supplementary Materials — A: search strategy. B: case descriptions. C: table of included studies. [file 4567192.f1.zip › 4567192.f1/Supplemental data file - seach strategy.docx]

| embase.com | 1360 | 1353 |
| --- | --- | --- |
| Medline ALL Ovid | 459 | 65 |
| Web of Science SCI-EXPANDED & SSCI | 457 | 191 |
| Cochrane CENTRAL register of Trials | 360 | 50 |
| Preliminary PubMed-search | 668 | 348 |
| **TOTAL** | **3304** | **2007** |

**embase.com 1360**

(((morphine/de OR opiate/de OR 'morphine derivative'/exp) AND ('intrathecal drug administration'/de OR 'intraspinal drug administration'/de OR 'spinal anesthesia'/de)) OR 'morphine'/dd_tl OR opiate/dd_tl OR ((morphine OR opiate* OR opioid* OR narcotic*) NEAR/6 (intrathecal* OR intra-thecal* OR spinal* OR cerebrospinal* OR intra-spinal* OR subarachnoid*)):ab,ti) AND ('adverse event'/de OR 'drug fatality'/de OR 'adverse drug reaction'/de OR 'respiration depression'/de OR hypoxia/exp OR hypercapnia/de OR somnolence/exp OR morbidity/de OR death/de OR fatality/de OR mortality/exp OR 'respiratory failure'/de OR 'respiratory arrest'/de OR intoxication/de OR 'drug overdose'/exp OR 'drug intoxication'/de OR (adverse OR ((respirat* OR CNS OR nervous-system*) NEAR/3 (depress* OR inhibition* OR effect* OR arrest* OR failure*)) OR hypoxi* OR hypercapni* OR somnolen* OR hypersomnolen* OR sleepiness* OR morbidit* OR death OR fatal* OR mortalit* OR ((severe OR serious) NEAR/3 side-effect*) OR intoxicat* OR overdos*):ab,ti) NOT (obstetrics/mj OR 'intrathecal pump'/mj OR 'malignant neoplasm'/exp/mj OR 'cesarean section'/exp/mj OR childbirth/exp/mj OR (obstetric* OR pump OR chronic* OR malign* OR cancer* OR cesarean* OR caesarea* OR c-sectio* OR labour* OR labor OR childbirth* OR child-birth* OR deliver*):ti) NOT (juvenile/exp NOT adult/exp) NOT ([animals]/lim NOT [humans]/lim) NOT [conference abstract]/lim NOT ((review/exp OR 'meta analysis'/de OR (review OR meta-analy*):ti) NOT ('case report'/de OR 'case study'/exp OR case*:ti))

**Medline ALL Ovid 459**

(((Morphine/ OR Opiate Alkaloids/) AND (Injections, Spinal / OR Anesthesia, Spinal/)) OR ((morphine OR opiate* OR opioid* OR narcotic*) ADJ6 (intrathecal* OR intra-thecal* OR spinal* OR cerebrospinal* OR intra-spinal* OR subarachnoid*)).ab,ti.) AND (exp Drug-Related Side Effects and Adverse Reactions/ OR Respiratory Insufficiency/ OR Hypoxia/ OR Hypercapnia/ OR Sleepiness/ OR Morbidity/ OR Death/ OR exp Mortality/ OR Drug Overdose/ OR (adverse OR ((respirat* OR CNS OR nervous-system*) ADJ3 (depress* OR inhibition* OR effect* OR arrest* OR failure*)) OR hypoxi* OR hypercapni* OR somnolen* OR hypersomnolen* OR sleepiness* OR morbidit* OR death OR fatal* OR mortalit* OR ((severe OR serious) ADJ3 side-effect*) OR intoxicat* OR overdos*).ab,ti.) NOT (*Obstetrics / OR *intrathecal pump/ OR exp * Neoplasms / OR * Cesarean Section / OR * Parturition / OR (obstetric* OR pump OR chronic* OR malign* OR cancer* OR cesarean* OR caesarea* OR c-sectio* OR labour* OR labor OR childbirth* OR child-birth* OR deliver*).ti.) NOT ((exp child/ OR exp infant/ OR pediatrics/ OR adolescent/) NOT exp adult/) NOT (exp animals/ NOT humans/) NOT ((review/ OR Systematic Review/ OR meta-analysis/ OR (review OR meta-analy*).ti.) NOT (case reports/ OR case*.ti.))

**Web of Science SCI-EXPANDED & SSCI 457**

TS=((((morphine OR opiate* OR opioid* OR narcotic*) NEAR/5 (intrathecal* OR intra-thecal* OR spinal* OR cerebrospinal* OR intra-spinal* OR subarachnoid*))) AND ((adverse OR ((respirat* OR CNS OR nervous-system*) NEAR/2 (depress* OR inhibition* OR effect* OR arrest* OR failure*)) OR hypoxi* OR hypercapni* OR somnolen* OR hypersomnolen* OR sleepiness* OR morbidit* OR death OR fatal* OR mortalit* OR ((severe OR serious) NEAR/2 side-effect*) OR intoxicat* OR overdos*))) NOT TI=((obstetric* OR pump OR chronic* OR malign* OR cancer* OR cesarean* OR caesarea* OR c-sectio* OR labour* OR labor OR childbirth* OR child-birth* OR deliver* OR child* OR infan* OR pediatr* OR paediatr* OR review* OR meta-analy* OR case*)) AND DT=(article)

**Cochrane CENTRAL register of Trials 360**

(((morphine OR opiate* OR opioid* OR narcotic*) NEAR/6 (intrathecal* OR intra NEXT thecal* OR spinal* OR cerebrospinal* OR intra NEXT spinal* OR subarachnoid*)):ab,ti) AND ((adverse OR ((respirat* OR CNS OR nervous NEXT system*) NEAR/3 (depress* OR inhibition* OR effect* OR arrest* OR failure*)) OR hypoxi* OR hypercapni* OR somnolen* OR hypersomnolen* OR sleepiness* OR morbidit* OR death OR fatal* OR mortalit* OR ((severe OR serious) NEAR/3 side NEXT effect*) OR intoxicat* OR overdos*):ab,ti) NOT ((obstetric* OR pump OR chronic* OR malign* OR cancer* OR cesarean* OR caesarea* OR c NEXT sectio* OR labour* OR labor OR childbirth* OR child NEXT birth* OR deliver*):ti)

**Preliminary PubMed-search 668**

(intrathecal OR spinal) AND (morphine) NOT (chronic) NOT (pump) NOT (caesarean section)
