# Supplementary material for: Serious Adverse Events after a Single Shot of Intrathecal Morphine: A Case Series and Systematic Review
Source: Pain Res Manag. 2022 Mar 10;2022:4567192. doi: 10.1155/2022/4567192 (PMC8930253; doi:10.1155/2022/4567192)
Supplement: Supplementary Materials — A: search strategy. B: case descriptions. C: table of included studies. [file 4567192.f1.zip › 4567192.f1/Supplemental data file - table studies.docx]

| First author | Age, gender | Type of surgery | Dose of morphine (mcg) | Concomitant administration of LA or sedatives? | Somnolence, Respiratory Depression or Hypotension | Life Threatening? | Treatment |
| --- | --- | --- | --- | --- | --- | --- | --- |
| Bernard^1^ |  | Spinal column | 300 |  | RD |  | N |
|  |  | Spinal column | 300 |  | RD |  | N |
|  |  | Spinal column | 300 |  | RD |  | N |
|  |  | Spinal column | 300 |  | RD |  | N |
| Bowrey^2^ |  | Orthopedic | 200 |  | RD, S |  | N |
| Chaney^3^ |  | Cardiac surgery | 4000 |  | S |  | - |
| Fitzpatrick^4^ |  | Cardiac surgery | 1000 |  | RD, S |  | N |
|  |  | Cardiac surgery | 2000 |  | RD, S |  | N |
| Gehling^5^ |  | Orthopedic | 100 | LA | RD |  | - |
|  |  | Orthopedic | 200 | LA | RD |  | - |
|  |  | Orthopedic | 200 | LA | RD |  | - |
| Gjessing^6^ | 73, F | Orthopedic | 2000 | LA | RD | Yes | - |
|  | 62, M | Orthopedic | 1400 | LA | RD |  | - |
|  | 72, F | Orthopedic | 1400 | LA | RD | Yes | N |
|  | 78, M | Orthopedic | 1200 | LA | RD | Yes | - |
|  | 85, F | Orthopedic | 1000 | LA | RD | Yes | N |
|  | 81, F | Orthopedic | 1000 | LA | RD | Yes | N |
|  | 82, F | Orthopedic | 1000 | LA | RD | Yes | N |
|  | 75, F | Abdominal surgery | 2000 | Sed | RD | Yes | - |
|  | 71, F | Abdominal surgery | 1200 | Sed | RD | Yes | N |
|  | 67, F | Abdominal surgery | 1000 | Sed | RD |  | - |
| Gray^7^ |  | Thoracic surgery | 800 |  | RD |  | N |
| Jun^8^ | 59, F | Abdominal surgery | 400 | Sed | RD, S | Yes | Mechanical Ventilation |
| Kalso^9^ | F | Orthopedic | 400 | LA, Sed | RD | Yes | - |
|  |  | Orthopedic | 400 | LA | RD |  | - |
| Samii^10^ |  |  | 1600 |  | RD |  | N |
|  |  |  | 1600 |  | RD |  | N |
| Sebel^11^ |  | Cardiac surgery | 4000 |  | RD |  | N |
| Shapiro^12^ | 72, F | Orthopedic | 200 | LA | RD, S |  | - |
| Suksompong^13^ | 69, F | Thoracic surgery | 300 | Sed | RD |  | N, Mechanical Ventilation |
| Wongyingsinn^14^ | 81 | Abdominal surgery | 150 | LA | RD |  | Ventilatory support |
|  | 75 | Abdominal surgery | 150 | LA | S |  | - |
| Akodjenou^15^ | 83, M | Abdominal surgery | 1000 | LA | RD | Yes | Mechanical Ventilation |
| Baskoff^16^ | 72, M | Abdominal surgery | 10000 | LA | RD, S | Yes | N, Mechanical Ventilation |
| Bicalho^17^ | 44, F | Abdominal surgery | 100 | LA, Sed | Diaphoresis |  | - |
| D'Oyley^18^ | 60, M | Abdominal surgery | 800 |  | RD |  | - |
| De All^19^ | 67, M | Abdominal surgery | 400 |  | Vertical nystagmus |  | N |
| De Gans^20^ | 61, M | Major vascular | 3000 |  | Meningitis |  | Antibiotics |
| De Morais^21^ | 45, M | Orthopedic | 4000 | LA | Diaphoresis |  | N |
| Dworzak^22^ | 72, F | Inguinal surgery | 100 | LA, Sed | RD, S | Yes | N, Flumazenil |
| Eran^23^ | 51, F | Thoracic surgery | 500 |  | S, PRES |  | - |
| Glass^24^ | 74, F | Orthopedic | 400 | LA, Sed | RD, H |  | N |
| Glynn^25^ | 71, M | Thoracic surgery | 5000 |  | S |  | N |
|  | 74, F | Abdominal surgery | 3000 |  | RD |  | N |
| Ip Yam^26^ | 76, M | Thoracic surgery | 2000 |  | RD, S, TIA | Yes | N |
| Kaiser^27^ | 81, M | Abdominal surgery | 5000 | LA | S |  | CSF-irrigation |
| Karpos^28^ | 27, F | Spinal column | 500 |  | Fascia dehiscence |  | Surgical treatment |
|  | 34, F | Spinal column | 9500 |  | Fascia dehiscence |  | Surgical treatment |
| King^29^ | 64, M | Abdominal surgery | 400 | LA | RD, S |  | N |
| Koning | 80, F | Abdominal surgery | 5000 | LA | RD, S |  | N |
|  | 72, M | Abdominal surgery | 4000 | LA, Sed | H |  | Norepinephrine |
|  | 74, F | Abdominal surgery | 5000 | LA, Sed | RD, S | Yes | N |
|  | 63, M | Abdominal surgery | 3000 | LA | RD, S | Yes | N |
| Korff^30^ | 59, F | Abdominal surgery | 600 | LA, Sed | Vertical nystagmus |  | - |
| Krenn^31^ | 72, F | Abdominal surgery | 100 | LA | RD, Nystagmus | Yes | N, minor effect |
| Liolios^32^ | 67, M | Abdominal surgery | 15000 |  | RD, S | Yes | N, mechanical ventilation |
| Lim^33^ | 74, M | Lower extremity | 100 | LA, Sed | S |  | N |
| Neustein^34^ | 73, F | Thoracic surgery | 250 |  | S |  | N |
| Odoom^35^ | 73, F | Major vascular | 3000 | Sed | RD |  | N |
|  | 82, M | Major vascular | 3000 | Sed | RD | Yes | N |
| Ong^36^ | 39, F | Abdominal surgery | 250 | LA, Sed | Pain |  |  |
| Paulus^37^ | 55, M | Inguinal surgery | 20,000 | LA, Sed | RD |  | N |
| Perrot^38^ | 42, M | Orthopedic | 8000 | Sed | RD, S | Yes | N |
| Pomonis^39^ | 90, F | Orthopedic | 15,000 | LA | RD, S | Yes | Nalorphine |
|  | 82, F | Orthopedic | 15,000 | LA | RD, S | Yes | Nalorphine |
|  | 61, M | Orthopedic | 15,000 | LA | RD, S | Yes | Nalorphine |
|  | 78, M | Orthopedic | 15,000 | LA | RD, S | Yes | Nalorphine |
| Rutili^40^ | 81, F | Abdominal surgery | 60 | LA, Sed | S |  | N |
| Ryan^41^ | 57, F | Orthopedic | 150 | LA, Sed | Hypothermia |  | Lorazepam |
| Scammell^42^ | 76, F | Orthopedic | 400 | LA, Sed | RD, S |  | N |
| Sidi^43^ | 40, M | Abdominal surgery | 4000 | S | RD, S | Yes | N, no effect |
| Vijayan^44^ | F | Orthopedic | “Inadvertantly large dose” | LA | S |  | N |

Abbreviations: H: Hypotension, F: Female, LA: Local Anesthetic, M: Male, mcg: microgram, N: naloxone, RD: Respiratory Depression, Sed: Sedative, S: somnolence. Missing data is left open. PRES: Posterior Reversible Encephalopathy Syndrome.

**Reference list:**

1 Bernard JM, Hommeril JL, Legendre MP, Passuti N, Pinaud M. Spinal or systemic analgesia after extensive spinal surgery: Comparison between intrathecal morphine and intravenous fentanyl plus clonidine. *J CLIN ANESTH* 1993; **5**: 231-6

2 Bowrey S, Hamer J, Bowler I, Symonds C, Hall JE. A comparison of 0.2 and 0.5 mg intrathecal morphine for postoperative analgesia after total knee replacement. *Anaesthesia* 2005; **60**: 449-52

3 Chaney MA, Smith KR, Barclay JC, Slogoff S. Large-dose intrathecal morphine for coronary artery bypass grafting. *ANESTH ANALG* 1996; **83**: 215-22

4 Fitzpatrick GJ, Moriarty DC. Intrathecal morphine in the management of pain following cardiac surgery. A comparison with morphine I.v. *BR J ANAESTH* 1988; **60**: 639-44

5 Gehling MH, Luesebrink T, Kulka PJ, Tryba M. The effective duration of analgesia after intrathecal morphine in patients without additional opioid analgesia: a randomized double-blind multicentre study on orthopaedic patients. *Eur J Anaesthesiol* 2009; **26**: 683-8

6 Gjessing J, Tomlin PJ. Postoperative pain control with intrathecal morphine. *ANAESTHESIA* 1981; **36**: 268-76

7 Gray JR, Fromme GA, Nauss LA. Intrathecal morphine for post-thoracotomy pain. *ANESTH ANALG* 1986; **65**: 873-6

8 Jun JH, Kim GS, Lee JJ, Ko JS, Kim SJ, Jeon PH. Comparison of intrathecal morphine and surgical-site infusion of ropivacaine as adjuncts to intravenous patient-controlled analgesia in living-donor kidney transplant recipients. *Singapore Med J* 2017; **58**: 666-73

9 Kalso E. Effects of intrathecal morphine, injected with bupivacaine, on pain after orthopaedic surgery. *BR J ANAESTH* 1983; **55**: 415-22

10 Samii K, Chauvin M, Viars P. Postoperative spinal analgesia with morphine. *BR J ANAESTH* 1981; **53**: 817-20

11 Sebel PS, Aun C, Fiolet J, Noonan K, Savege TM, Colvin MP. Endocrinological effects of intrathecal morphine. *Eur J Anaesthesiol* 1985; **2**: 291-6

12 Shapiro A, Zohar E, Zaslansky R, Hoppenstein D, Shabat S, Fredman B. The frequency and timing of respiratory depression in 1524 postoperative patients treated with systemic or neuraxial morphine. *J Clin Anesth* 2005; **17**: 537-42

13 Suksompong S, Pongpayuha P, Lertpaitoonpan W, Von Bormann B, Phanchaipetch T, Sanansilp V. Low-dose spinal morphine for post-thoracotomy pain: A prospective randomized study. *J Cardiothorac Vasc Anesth* 2013; **27**: 417-22

14 Wongyingsinn M, Baldini G, Stein B, Charlebois P, Liberman S, Carli F. Spinal analgesia for laparoscopic colonic resection using an enhanced recovery after surgery programme: Better analgesia, but no benefits on postoperative recovery: A randomized controlled trial. *Br J Anaesth* 2012; **108**: 850-6

15 Akodjenou J, Mewanou S, Ahounou E, Zoumenou E. Severe morbidity and death associated with drug errors while performing spinal anesthesia. *J Clin Anesth* 2019; **58**: 48-9

16 Baskoff JD, Watson RL, Muldoon SM. Respiratory arrest after intrathecal morphine. A case report. *ANESTHESIOL REV* 1980; **7**: 12-5

17 Bicalho GP, Viana Castro CH, Cunha Cruvinel MG, Bessa Jr RC. Excessive sweating and hypothermia after spinal morphine. Case report. *Rev Bras Anestesiol* 2006; **56**: 52-6

18 D'Oyley DA, McDonald NJ. Intrathecal morphine and intravenous remifentanil analgesia for a patient undergoing hepatic resection surgery. *Can J Anaesth* 2008; **55**: 254-5

19 De All J, Repetto MF, Tagliapietra V, Risso J, Chirio F, Gnocchi C. [Vertigo and vertical nystagmus associated with intrathecal morphine administration and resolution by naloxone]. *Medicina* 2011; **71**: 457-8

20 de Gans J, van der Heide H. [Purulent meningitis caused by Pseudomonas aeruginosa following an operation of the aortic bifurcation, presumably related to intrathecal analgesia]. *Nederlands tijdschrift voor geneeskunde* 1983; **127**: 377-81

21 De Morais BS, Silva YP, Cruvinel MGC, De Castro CHV, Hermeto MV. Accidental subarachnoid administration of 4 mg of morphine. Case report. *Rev Bras Anestesiol* 2008; **58**: 160-4

22 Dworzak H, Fuss F, Buttner T. [Persisting respiratory depression following intrathecal administration of morphine and simultaneous sedation with midazolam]. *Anaesthesist* 1999; **48**: 639-41

23 Eran A, Barak M. Posterior reversible encephalopathy syndrome after combined general and spinal anesthesia with intrathecal morphine. *Anesth Analg* 2009; **108**: 609-12

24 Glass PSA. Respiratory depression following only 0.4 mg of intrathecal morphine. *ANESTHESIOLOGY* 1984; **60**: 256-7

25 Glynn CJ, Mather LE, Cousins MJ. Spinal narcotics and respiratory depression. *LANCET* 1979; **2**: 356-7

26 Ip Yam C, Salt J. Transient ischaemic attack following carbon dioxide retention due to intrathecal morphine. *Anaesthesia* 1992; **47**: 1098

27 Kaiser KG, Bainton CR. Treatment of intrathecal morphine overdose by aspiration of cerebrospinal fluid. *ANESTH ANALG* 1987; **66**: 475-7

28 Karpos PA, Jones CK, McNamara MJ, Spengler DM. Persistent leakage of cerebrospinal fluid after intrathecal administration of morphine in an operation on the lumbar spine. A report of two cases. *The Journal of bone and joint surgery American volume* 1994; **76**: 916-8

29 King HK, Tsai SK. Delayed respiratory depression following repeated intrathecal low dose morphine. *ANAESTH INTENSIVE CARE* 1985; **13**: 334-5

30 Korff C, Peter M, Burkhard PR. Downbeat nystagmus as a manifestation of intrathecal morphine toxicity. *Eur J Anaesthesiol* 2007; **24**: 201-2

31 Krenn H, Jellinek H, Haumer H, Oczenski W, Fitzgerald R. Naloxone-resistant respiratory depression and neurological eye symptoms after intrathecal morphine. *Anesth Analg* 2000; **91**: 432-3

32 Liolios A, Andersen FH. Selective spinal analgesia. *Lancet* 1979; **2**: 357

33 Lim PC, Macintyre PE. An audit of intrathecal morphine analgesia for non-obstetric postsurgical patients in an adult tertiary hospital. *Anaesthesia and intensive care* 2006; **34**: 776-81

34 Neustein SM, Cottone TM. Prolonged respiratory depression after intrathecal morphine. *J Cardiothorac Vasc Anesth* 2003; **17**: 230-1

35 Odoom JA, Sih IL. Respiratory depression after intrathecal morphine. *ANESTH ANALG* 1982; **61**: 70

36 Ong B, Baker C. Temporary back and leg pain after bupivacaine and morphine spinal anaesthesia. *Can J Anaesth* 1995; **42**: 805-7

37 Paulus DA, Paul WL, Munson ES. Neurologic depression after intrathecal morphine. *ANESTHESIOLOGY* 1981; **54**: 517-8

38 Perrot G, Muller A, Laugner B. [Accidental overdose of intrathecal morphine. Treatment with intravenous naloxone alone]. *Annales francaises d'anesthesie et de reanimation* 1983; **2**: 412-4

39 Pomonis SP, Economacos G, Costopanajiocoy SP. Overdose of intrathecal morphine. *Anaesthesia* 1986; **41**: 670

40 Rutili A, Maggiani M, Bertelloni C, Molinari D. Persistent overdose caused by a very small dose of intrathecal morphine in an elderly patient undergoing vaginal hysterectomy: A case report. *Minerva Anestesiol* 2007; **73**: 433-6

41 Ryan KF, Price JW, Warriner CB, Choi PT. Persistent hypothermia after intrathecal morphine: case report and literature review. *Can J Anaesth* 2012; **59**: 384-8

42 Scammell SJ. Apnoea with intrathecal morphine. *Anaesthesia and intensive care* 2000; **28**: 708

43 Sidi A, Davidson JT, Behar M, Olshwang D. Spinal narcotics and central nervous system depression. *Anaesthesia* 1981; **36**: 1044-7

44 Vijayan R, Chan L, Raveenthiran R. Continuous spinal anaesthesia--early experience in University Hospital, Kuala Lumpur. *Med J Malaysia* 1995; **50**: 401-10
